# What is the normal value of the neutrophil-to-lymphocyte ratio?

**DOI:** 10.1186/s13104-016-2335-5

**Published:** 2017-01-03

**Authors:** Patrice Forget, Céline Khalifa, Jean-Philippe Defour, Dominique Latinne, Marie-Cécile Van Pel, Marc De Kock

**Affiliations:** 1 Department of Anesthesiology, Cliniques Universitaires Saint-Luc, Institute of Neuroscience (pole CEMO), Université Catholique de Louvain, 1200 Brussels, Belgium; 2 Department of Clinical Biology, Cliniques Universitaires Saint-Luc, 1200 Brussels, Belgium; 3 CESI–Prévention et Protection ASBL, Brussels, Belgium; 4 Department of Anesthesiology and Perioperative Medicine, Vrije Universiteit Brussel (VUB), Universitair Ziekenhuis Brussel (UZ Brussel), Laarbeeklaan, 101, 1090 Brussels, Belgium

**Keywords:** Neutrophil-to-lymphocyte ratio, Cells count, Laboratory testing, Reference values

## Abstract

**Background:**

Neutrophil-to-lymphocyte ratio (NLR) has proven its prognostic value in cardiovascular diseases, infections, inflammatory diseases and in several types of cancers. However, no cut-off has been proposed on the basis of reference values coming from healthy population.

**Methods:**

Routine blood samples were obtained (n = 413) from workers (age: median 38, range: 21–66 years) involved in a health care prevention program, to determine means, standard deviations (SDs), 95% confidence intervals (95% CI), percentiles P2.5 and P97.5. A second independent sample of healthy volunteers is compared (n = 29).

**Results:**

The mean NLR is 1.65 [±1.96 SD: 0.78–3.53] (95% CI [0.75–0.81] and [3.40–3.66]). In the second cohort (healthy control), the NLR values are in the same range, whichever the used analyzer. No NLR assessed in the validation series is out of the proposed limits.

**Conclusions:**

We have identified that the normal NLR values in an adult, non-geriatric, population in good health are between 0.78 and 3.53. These data will help to define the normal values of the NLR.

**Electronic supplementary material:**

The online version of this article (doi:10.1186/s13104-016-2335-5) contains supplementary material, which is available to authorized users.

## Background

Neutrophil-to-lymphocyte ratio (NLR) is a simple parameter to assess easily the inflammatory status of a subject. It has proven its usefulness in the stratification of mortality in major cardiac events [1, 2], as a strong prognostic factor in several types of cancers [3–10], or as a predictor and a marker of inflammatory or infectious pathologies (such is pediatric appendicitis) and postoperative complications [11, 12].

However, which NLR value is correlated with a higher risk for these patients? Which cut-off value, for the NLR, will discriminate normal from abnormal results?

Different values of NLR, with different methods, in different populations (cancerous or not) are cited in the literature. And finally, no universal value is currently available. Therefore, there is a need for reference values to progress in the use of this marker.

The aim of this study is to determine the limits of the values of the NLR that are observed in an adult, non-geriatric population, without any acute illness and/or chronic debilitating disease.

## Methods

### Subjects and data collection

With the agreement of the Ethical Committee (ref. 2014/451, Chairperson: Prof. Jean-Marie Maloteaux, av. Hippocrate, 55-14, 1200 Brussels), we performed the following analyses. Blood samples for routine control between October 2011 and 2012 were identified from 413 participants of the health care prevention program for workers. Considering the analysis as retrospective, given the fact that the samples were pre-collected and treated anonymously, the Ethical Committee gave a waiver for individual consent. Consequently, no written informed consent for participation in the study was obtained from participants.

Median age was 38 years (range: 21–66 years). Typically, these subjects were in good health, permitting a full time job without restriction, and without any active disease, including cancer and infectious disease and are representative of the population as a whole. Blood samples were obtained for routine hematologic control in workers potentially exposed to X-rays or any others ionizing radiation. Samples were treated anonymously, including sex and date of birth. No information was therefore available about habits (e.g. tobacco use) and possible comorbidities (e.g. obesity or vascular disease).

### Normal controls

Our goal is to identify the higher boundary observed in a population in good health, but not excluding non-debilitating disease, tobacco use or oral contraception. Therefore, it would be important to compare the results to those obtained in a second, and independent, sample (n = 29) of normal controls, strictly selected on the basis of perfect health, coming from an historical cohort. This cohort was used during the process of the calibration of the blood analyser. The subjects in this second cohort were in the same range of age, but, after interrogation and examination, carefully selected on the basis of the absence of any chronic disease or substance/medications (abuse, including tobacco use).

### Samples analyses

Anticoagulated whole blood from routine controls were processed on Sysmex XE2100 [TOA Medical Electronics, Kobe, Japan], Normal controls were analysed on Sysmex XN2000 (TOA Medical electronics Co, Kobe, Japan), Advia 2120 (Bayer Diagnostics, Tarritown, NY, USA), DXH800 (Beckman Coulter, Miami, FL USA), Cell-Dyn Sapphire (Abbott Diagnostics Santa Clara, CA, USA) for the determination of the complete blood cell counts and differential counts of leukocytes. The absolute neutrophil count was divided by the absolute lymphocyte count to calculate the NLR.

### Statistical analyses

For the statistical analyses, laboratory parameters fit a log-normal distribution. Logarithmic conversion was therefore used for calculating means and 95% confidence limits (95% CI) are presented as arithmetic mean ± 1.96 SD (with their 95% CI). Power analysis shows that, to test the hypothesis that <2.5% of the normal controls would be out of the proposed limits, 28 subjects are needed. It is the reason why we included 29 blood samples of the historical cohort for the external validation.

All the analyses were performed with STATISTICA (data analysis software system, version 7, Statsoft Inc. 2004, Tulsa).

## Results

Results coming from the blood samples of the 413 subjects of the main cohort are presented in Table 1. Briefly, the mean NLR is 1.65 [±1.96 SD: 0.78–3.53] (95% CI [0.75–0.81] and [3.40–3.66]). In the second cohort (healthy control), the NLR values are in the same range, whichever the used analyzer (Table 1). Consequently, no NLR assessed in the validation series is out of the proposed limits.

**Table 1:** Neutrophil-to-lymphocyte ratios obtained in two cohort of subjects (n = 413 and n = 29) on five blood analysers, expressed in mean, SD and lower/upper limits of the mean ± 1.96 SD range (and their 95% CI)

	Mean	SD	Lower limit	95% CI		Upper limit	95% CI	
Initial cohort (n = 413)								
Sysmex XE2100	1.65	1.47	0.78	0.75	0.81	3.53	3.40	3.66
Control cohort (n = 29)								
Beckman DXH800	1.76	1.42	0.89	0.78	1.02	3.49	3.06	3.97
Siemens 2120i	1.86	1.39	0.97	0.86	1.10	3.54	3.13	4.00
Abbott SAPHYR	1.68	1.42	0.85	0.74	0.96	3.32	2.92	3.79
Sysmex XN2000	1.69	1.37	0.91	0.81	1.03	3.12	2.77	3.52

NLR sample stability over 48 h was assessed; there was no significant variation over this time period and the NLR was then considered stable.

## Discussion

We show here that the NLR values, in a sample of 413 active subjects in good health, are between 0.78 and 3.58. We confirm that no normal control (selected on the basis of the absence of any comorbidities and substance abuse, including tobacco) exceeded this range.

There are a lot of examples in the literature of the interest of the NLR as an independent prognostic factor of morbidity and mortality in several conditions, such as cancers and cardiovascular diseases. NLR is also useful in the prediction and the detection of inflammatory and infectious conditions, and their postoperative complications [11, 12]. Nevertheless, none of these studies based their cut-off on data coming from population in good health, and none on data coming from normal controls. Some of these studies chose their cut-off value on the basis of the median, higher quartile or values determined by the use of receiver-operating curves (to predict the occurrence of a condition, e.g. cancer recurrence of infection) [13–15].

In contrast with these studies, we found here limits based on a sample of an active adult population in good health.

This value can be used as a cut-off to differentiate patients that are in the range of a population in good health or not [16]. Can it be used in other populations that this from which this sample is coming? Age is certainly a limit, as we tested only adult, non-geriatric, subjects (between 21 and 66 years). Additionally, we did not have the complete documentation of substance use/abuse, like tobacco or oral contraceptive. Ethnic group origins, sex and season of assessment have not been included in the analyses. However, in the sample of normal control, we see that the 95% CI permits to say that it is very improbable that normal controls will have a NLR value of 3.5. Nevertheless, it seems logical to interpret cautiously this type of parameter (like any other), in a clinical context to adjust the interpretation, even if based on data coming from a comparable population (Additional file 1).

## Conclusions

We have identified that normal NLR values in an adult, non-geriatric, population in good health are between 0.78 and 3.53. These data may help the researcher as the clinician searching for a cut-off for the NLR, until now lacking.

## Additional file

**Additional file 1.** Raw data of neutrophil-to-lymphocyte ratio.

## Abbreviations

SDs: standard deviations
95% CI: 95% confidence intervals
P2.5 and P95.5: percentiles 2.5 and 97.5%
NLR: neutrophil-to-lymphocyte ratio
